# Chromatin accessibility directly governs flavonoid biosynthesis and indirectly orchestrates cannabinoid production in *Cannabis*

**DOI:** 10.3389/fpls.2025.1687700

**Published:** 2026-01-19

**Authors:** Yuanchang Ma, Xiuye Wei, Weixin Zhou, An Xie, Yongzhong Chen, Chen Yang, Lingcheng Chen, Linlin Dong, Kang Ning

**Affiliations:** 1 Key Laboratory of Specialty Agri-product Quality and Hazard Controlling Technology of Zhejiang Province, College of Life Sciences, China Jiliang University, Hangzhou, China; 2 Key Laboratory of Beijing for Identification and Safety Evaluation of Chinese Medicine, Institute of Chinese Materia Medica, China Academy of Chinese Medical Sciences, Beijing, China; 3 School of Pharmacy, Chengdu University of Traditional Chinese Medicine, Chengdu, China

**Keywords:** cannabis, chromatin accessibility, secondary metabolites, cannabinoids, flavonoid

## Abstract

Secondary metabolites in hemp enhance its pharmaceutical and nutraceutical value, yet the epigenetic regulatory network underlying secondary metabolite biosynthesis remains poorly understood in hemp. Here, we profiled the inflorescences of two cultivars with different trichome density by integrating metabolomics, transcriptomics and ATAC-seq. Multi-omics data revealed pronounced differences in metabolites (491 differentially accumulated metabolites (DAMs)), transcripts (8343 differentially expressed genes (DEGs)), and chromatin accessibility (11376 different accessibility genes, (DAGs)) between two cultivars. Integrated analyses reveal that increased chromatin accessibility at the promoters of several flavonoid-biosynthetic genes up-regulated their expression, resulting in the accumulation of flavonoids. Although chromatin accessibility of cannabinoid biosynthetic gene promoters modulates content, differential chromatin accessibility of the promoter of fatty acid biosynthetic and trichome density (trichome initiation, MeJA signaling, and identity of floral organ) related genes constitutes the key driver underlying cannabinoid divergence between two cultivars. Our study advances the understanding of epigenetic regulation of plant secondary metabolites and offers a novel strategy for enhancing cannabinoid and flavonoid content in Cannabis, providing efficient and precise molecular markers for the selection and breeding of new cannabis varieties.

## 1 Introduction

Hemp (*Cannabis sativa* L.) is a versatile dioecious plant belonging to the *Cannabaceae* family, which has been cultivated for thousands of years for its many applications in agriculture, medicine, and industry (Xie et al., 2023). Characterized by its serrated leaves, dioecious flowering structure, and adaptability to different climates, fibrous stalks for textiles, and its seeds as a source of nutrients, cannabis has always been valued. In recent decades, however, there has been a shift in scientific and commercial interest in the abundant secondary metabolites of cannabis, particularly cannabinoids (e.g., Δ^9^-tetrahydrocannabinol (THC) and cannabidiol (CBD)), terpenoids, and flavonoids (Jin et al., 2020). These compounds consolidate the pharmacological potential of cannabis to provide therapeutic benefits for disorders such as chronic pain, epilepsy, and anxiety, while also driving the therapeutic and economic value of cannabis in the global pharmaceutical market (Gonçalves et al., 2019).

The surface of the female inflorescences of hemp bears a specialized organ—the capitate-stalked trichome—which functions as a biosynthetic factory for cannabinoids (Tanney et al., 2021; Wang et al., 2021; Xie et al., 2023). Studies have revealed that cannabinoids are predominantly enriched in the female inflorescences, which also contain abundant terpenoids and flavonoids. Leaves, stems, and roots additionally accumulate secondary metabolites such as sterols and triterpenoids (Jin et al., 2020). This metabolome-wide diversity is clinically considered to confer an “entourage effect”. The biosynthetic pathway for cannabinoids has been fully elucidated. Cannabinoids originate from the condensation of olivetolic acid (a polyketide) and geranyl pyrophosphate (a terpenoid precursor) catalyzed by cannabigerolic acid synthase (CBGAS). Subsequent oxidations by THCA synthase (THCAS) or CBDA synthase (CBDAS) yield the corresponding acidic and neutral cannabinoids (Jin et al., 2020). Likewise, the pathways for terpenoid and flavonoid biosynthesis are well mapped. While these biosynthesis pathways are well-characterized, the upstream regulatory networks remain underexplored. For instance, cannabinoid synthesis is modulated by light; different LED spectra not only influence flowering time but also alter cannabinoid content and ratios (Namdar et al., 2019). In addition, the plant-associated microbiome can modulate the production of secondary metabolites (Taghinasab and Jabaji, 2020).

Chromatin exists in two states, determined jointly by nucleosome positioning and histone modifications: a highly compact, condensed structure and a loose, open conformation (Felsenfeld et al., 1996). In the open state, chromatin facilitates access of transcription factors and RNA polymerase to DNA, thereby activating transcription. Recent advances highlight ATAC-seq as a powerful tool to map chromatin accessibility landscapes and identify regulatory elements (e.g., enhancers, promoters) in plant development, biotic and abiotic stress resistance (Li et al., 2019). For example, Zheng et al. (2025) employed ATAC-seq to chart the chromatin-accessibility landscape of early maize seed development, uncovering a positive correlation between promoter accessibility and gene expression and identifying stage-specific transcription-factor footprints. Li et al. (2024) integrated ATAC-seq with RNA-seq to dissect apple fruit development and elucidated the molecular mechanism by which Dof transcription factors regulate malate and sugar accumulation. During rice lemma development, the accessibility of open chromatin regions (OCRs) positively correlates with the expression of adjacent genes; notably, chromatin openness upstream of BZR1 and SCL6-IIb is a key determinant of grain shape (Chen et al., 2025). Furthermore, environmental stressors may induce chromatin reconfiguration. Significant changes in chromatin accessibility were found in tea plants under cold stress, with 66 cold-induced transposase hypersensitive sites (THSs). Chromatin accessibility is also altered in rice under heat stress (Qiu et al., 2023). Upon BmNPV infection, host chromatin undergoes progressive remodeling (Kong et al., 2020). Metabolic pathways in tomato fruits during senescence can be influenced by chromatin remodeling (Guo et al., 2023). The application of nanomaterials can also alter the chromatin accessibility of plants (Ning et al., 2024b).

Constrained by strict regulations and a complex genetic background, molecular research on industrial hemp remains in its infancy. Although a reference genome has been published, the cannabinoid biosynthetic pathway has been elucidated, organ-specific metabolite profiles have been mapped by metabolomics, and several transcriptional regulators have been identified, systematic investigation of epigenetic regulation is still scarce. Omics technologies can generate a vast amount of data, while the integration of multiple omics can systematically dissect the regulatory networks underlying plant trait formation. The combination of metabolomics, transcriptomics and ATAC-seq can elucidate the biosynthetic mechanisms of plant secondary metabolites across the DNA-RNA-metabolite levels. In this study, we employed the inflorescences of high-glandular-trichome (H) and low-glandular-trichome (O) cultivars as a model. Integrating metabolomics, transcriptomics, and ATAC-seq, we comprehensively delineated metabolic, transcriptional, and chromatin-accessibility differences between the two cultivars. Joint multi-omics analyses further pinpointed key regulatory modules in which dynamic promoter accessibility governs the expression of structural genes, ultimately driving substantial divergence in flavonoid and terpenoid contents. These findings lay a theoretical foundation for epigenetic improvement of industrial hemp. This study bridges the gap between chromatin biology and phytochemistry, offering a comprehensive framework to decode the epigenetic drivers of hemp’s biochemical richness.

## 2 Materials and methods

### 2.1 Plant materials

Two industrial hemp cultivars, designated as “H” (high-glandular-trichome phenotype, with abundant trichomes distributed in sepals and leaves) and “O” (low-glandular-trichome phenotype, lacking trichomes in sepals and leaves), were cultivated under open-field conditions at Kunming, Yunnan Province, China. Plants were grown under natural sunlight and standard agricultural practices (Ouyang et al., 2025). When the H variety reached its flowering stage (at this point, the O variety has only a few inflorescence, and the O variety has only a few inflorescence throughout its entire life cycle), three phenotypically uniform plants with synchronized inflorescence development were selected from each cultivar. For each biological replicate, 4 grams of female inflorescences were collected, immediately flash-frozen in liquid nitrogen, and divided into three aliquots. All samples were stored at -80 °C for further use.

### 2.2 Extraction and LC-MS/MS for untargeted metabolomic analysis

Frozen samples were homogenized in 0.5 mL ice-cold 80%methanol, incubated at 20°C for 30 min, and centrifuged at 20,000×g for 15 min at 4°C. The supernatants were vacuum-dried, reconstituted in 100μL 80%methanol, and stored at 80°C for LC-MS analysis. Quality control was ensured by pooling 10 μL of each supernatant to create QC samples, minimizing technical variability (Ning et al., 2024a; Sawada and Hirai, 2013).

Raw data were converted to mzXML format and processed through the XCMS, CAMERA, and metaX toolboxes in R (version 4.0.0) for data pretreatment, including peak detection, retention time correction, peak alignment, and annotation of isotopes and adducts. A three-dimensional matrix was constructed with retention time (RT)-m/z pairs as peak indices, sample names as observations, and ion intensities as variables. Metabolites were annotated by matching experimental m/z values to the KEGG and HMDB databases (mass tolerance <10 ppm), followed by molecular formula validation using isotopic distribution patterns and an in-house fragment spectrum library. Statistical analyses involved median-normalized protein intensities, hierarchical clustering (pheatmap), PCA (metaX), and PLS-DA (ropls) with VIP scores. Significantly altered metabolites were filtered using combined thresholds (p < 0.05, fold change >1.2, VIP >1). Pathway enrichment analysis was conducted via hypergeometric tests and GSEA (v4.1.0) with MSigDB, applying significance criteria (|NES| >1, NOM p < 0.05, FDR q < 0.25) (Ning et al., 2022). Metabolic networks were visualized based on KEGG pathway annotations.

### 2.3 RNA-seq and analysis

Total RNAs were extracted by TRIzol (Huayueyang, Beijing). The samples were ground into powder under liquid nitrogen conditions, and the total RNA was extracted using the EZNA Plant RNA Kit (Omega, Shanghai, China). The RNA was used to construct libraries, which were then sequenced on an Illumina HiSeq2500 as demonstrated by Fang et al.

The raw sequencing data were filtered and trimmed using SeqPrep (https://github.com/jstjohn/SeqPrep) and sickle (https://github.com/najoshi/sickle). Then the clean data were aligned to the *Cannabis sativa* reference genome (cs10, https://www.ncbi.nlm.nih.gov/datasets/genome/GCA_900626175.2/) using Bowtie2 software (v2.5.4, http://bowtie-bio.sourceforge.net/bowtie2/manual.shtml). Differential expression analysis was performed using the DESeq2 package (v1.38.0) in R(v4.2.1). Raw count data were normalized using the DESeq2 internal normalization method. Genes with low read counts (mean CPM < 1 across all samples) were filtered out. Differentially expressed genes (DEGs) were identified, with adjusted p -values calculated. Genes with |log2 fold change| ≥ 1 and adjusted p -value < 0.05 were considered significantly differentially expressed. The Gene Ontology (GO) functional enrichment and Kyoto Encyclopedia of Genes and Genomes ontology (KO) pathway analysis were carried out using Goatools (https://github.com/tanghaibao/Goatools) (Ning et al., 2021).

### 2.4 ATAC-seq and analysis

The samples were ground into a fine powder under liquid nitrogen. The nuclei were then suspended in a transposition reaction system containing Tn5 transposase (YEASEN, Shanghai, China). DNA was treated as follows: incubated at 55 °C for 5 min with 0.2% SDS, treated with 1.8× AMPure XP beads at room temperature for 5 min, washed twice with 80% ethanol and dried at room temperature for 3 min and eluted with 10 mM Tris-HCl (pH 8.0). The resulting product was subjected to PCR amplification with the introduction of specific indexes. Library fragments of approximately 200-700bp were obtained through bead selection. The concentration of the library was measured using Qubit (Thermo, Qubit3.0, USA) and the integrity of the fragments was assessed using a Bioanalyzer 2100 (Agilent, CA, USA). Perform PE150 (Paired-End 150 bp) sequencing on the Illumina NovaSeqXP platform following the standard protocol (Smith et al., 2021).

The raw paired-end reads were trimmed and quality controlled using Trimmomatic (v0.36, http://www.usadellab.org/cms/?page= trimmomatic) with parameters (SLIDINGWINDOW: 4:15 MINLEN: 75). Clean reads were aligned separately with the reference genome with orientation mode by using bowtie2 (v2.5.0, http://bowtie-bio.sourceforge.net/bowtie2/manual.shtml). A dynamic Poisson distribution was used to calculate the p-value of the specific region based on the unique mapped reads by MACS3 (v2.2.9.1) software. A region with p-value < le−03 was defined as a peak. The markdup function of the Sambamba software (v1.0.1) was used to mark and remove PCR duplicate reads. The IDR (v2.0.3) method is used to extract highly consistent peak regions from multiple biological replicates, ensuring the reliability of the results. For differential peak analysis, DiffBind (v3.8.4) is employed to detect differential binding regions under different experimental conditions. DARs (different accessibility regions) were identified with a threshold of |log2FoldChange|≥1 and q<0.05. Functionally annotated via Gene Ontology (GO) and KEGG pathway enrichment analyses using hypergeometric testing (p <0.05), following established protocols.

### 2.5 QPCR

RNA used for transcriptome was used for QPCR. cDNA was synthesized using the FastQuant RT Kit (Tiangen, China). Each gene was analysed in three biological replicates and three technical replicates. The *CsUBQ* (*LOC115724769*) gene was used as an internal reference to normalize gene expression data (Zhou et al., 2025). Fold change was calculated using the 2^−ΔΔCt^ method (Livak and Schmittgen, 2001). Details regarding the QPCR primers are listed in **Supplementary Table S1**.

### 2.6 Gene set enrichment analysis

GSEA was conducted using GSEA software (v4.2.3 http://www.gsea-msigdb.org/gsea/login.jsp). A pre-ranked gene list was generated by ranking all expressed genes by signed LFC × -log10(p-value), derived from DESeq2 results. Enriched pathways with FDR q < 0.25 were subjected to leading edge analysis to identify core genes driving enrichment. Pathway results were cross-validated using clusterProfiler v4.6.0 for GO/KEGG enrichment (FDR < 0.05) and fgsea v1.20.0 for rapid GSEA with 10,000 permutations to confirm consistency (Mootha et al., 2003; Subramanian et al., 2005).

## 3 Results and discussion

### 3.1 Metabolomics profiling of inflorescence of two hemp cultivars

**Figure 1:**
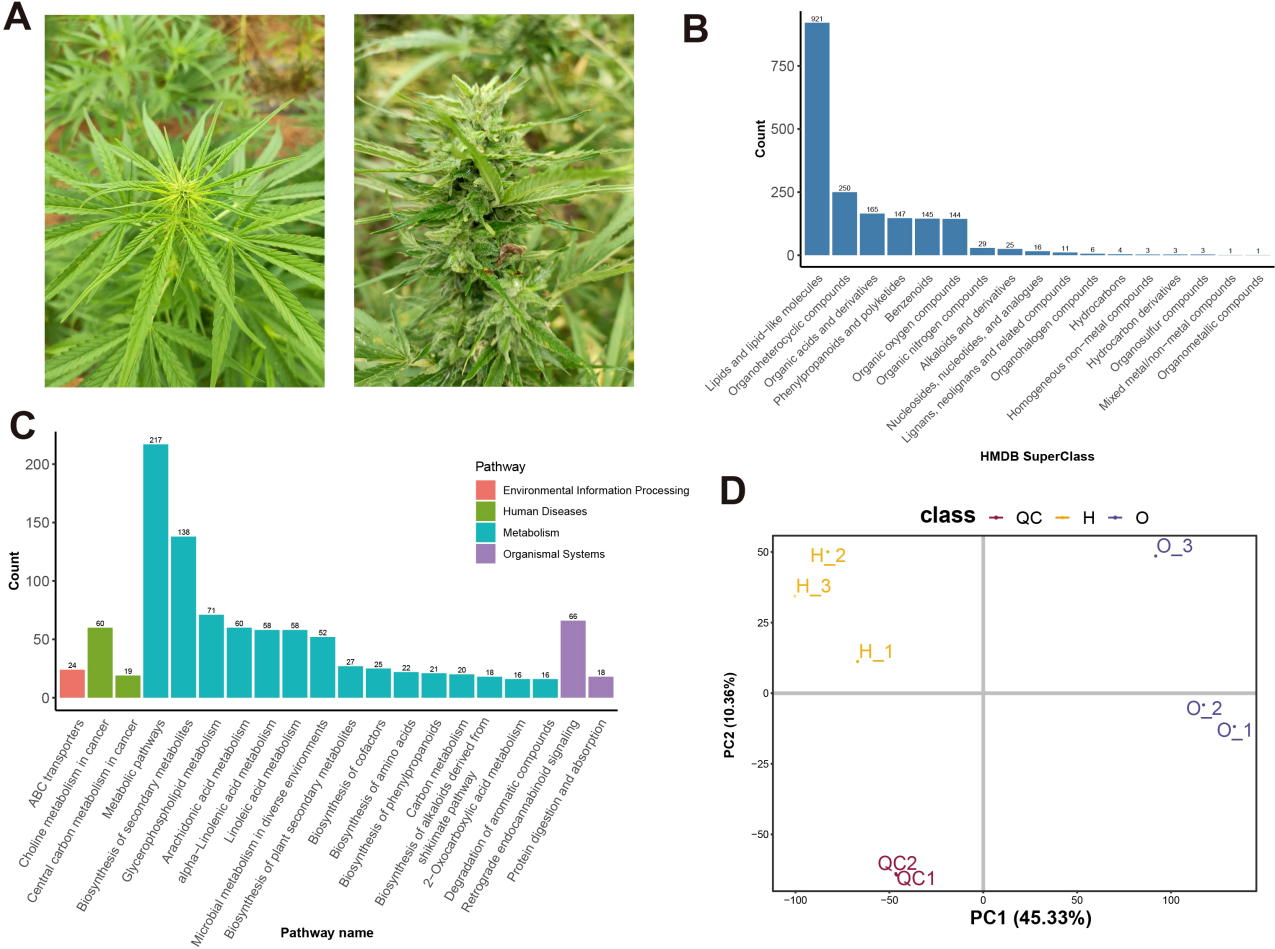
Metabolomics Profiling of two hemp cultivars. **(A)** The appearance of the O cultivar (left) and the H cultivar (right). Annotation of Metabolites based on HMDB database **(B)** and KEGG database **(C)**. **(D)** Principal component analysis (PCA) of metabolomic data.

Hemp is rich in a variety of metabolites, with differences in the content and types of metabolites across different organs. The female inflorescences of cannabis are the primary organ for the biosynthesis of cannabinoids (Xie et al., 2023). More specifically, the leaves and sepals in the female inflorescences are covered with a large number of stalked glandular trichomes. Glandular trichomes consist of secretory disc cells and secretory cavity, where secondary metabolites (including cannabinoids and terpenes) are produced and stored (Hancock et al., 2024; Livingston et al., 2020; Tanney et al., 2021). In this study, two cultivars with significant morphological differences were selected. The inflorescences of ‘H’ cultivar are densely covered with glandular trichomes, while those of ‘O’ cultivar have only a small number of glandular trichomes (**Figure 1A**).

Given the significant morphological differences between the two cultivars, we speculate that there are substantial differences in the metabolite profiles of their inflorescences. Therefore, we utilized non-targeted metabolomics to characterize the metabolite profiles of the two cultivars. Totally, 2153 metabolites were identified, including 921 Lipids and lipid-like molecules, 250 Organoheterocyclic compounds, 165 Organic acids and derivatives, 147 Phenylpropanoids and polyketides, and 145 Benzenoids (**Figure 1B**; **Supplementary Table S2**). Cannabinoids like Cannabichromene, delta8-Tetrahydrocannabinol were also detected in metabolomic data. The Cannabis inflorescence, a heterogeneous assemblage of organs—leaves and flowers—at various developmental stages, is highly abundant in metabolites (Kotiranta et al., 2024; Mahlberg and Kim, 2004). In cultivar H, bracts and leaf surfaces are densely covered with glandular trichomes that accumulate a broad spectrum of secondary metabolites. Concurrently, the inflorescence contains seeds at different stages; these seeds themselves are rich in lipids and secondary metabolites like flavanones (Ning et al., 2022). This seed-derived lipid pool likely accounts for the detection of as many as 921 distinct lipid species in our metabolomic dataset. Then the metabolites were annotated to the KEGG pathway. Clycerophospholipid metabolism (71), arachidonic acid metabolism (60), alpha-linolenic acid metabolism (58), and linolenic acid metabolism (58) pathways metabolites were identified (**Figure 1C**).

Principal component analysis (PCA) showed a clear separation according to cultivar PC1 explains 45.33% of the variance, and PC2 explains 10.36% of the variance. Together, these two principal components explain 55.69% of the variance (**Figure 1D**), indicating that these two principal components can represent the variability of the original data well.

### 3.2 Different accumulated metabolites between the two cultivars

**Figure 2:**
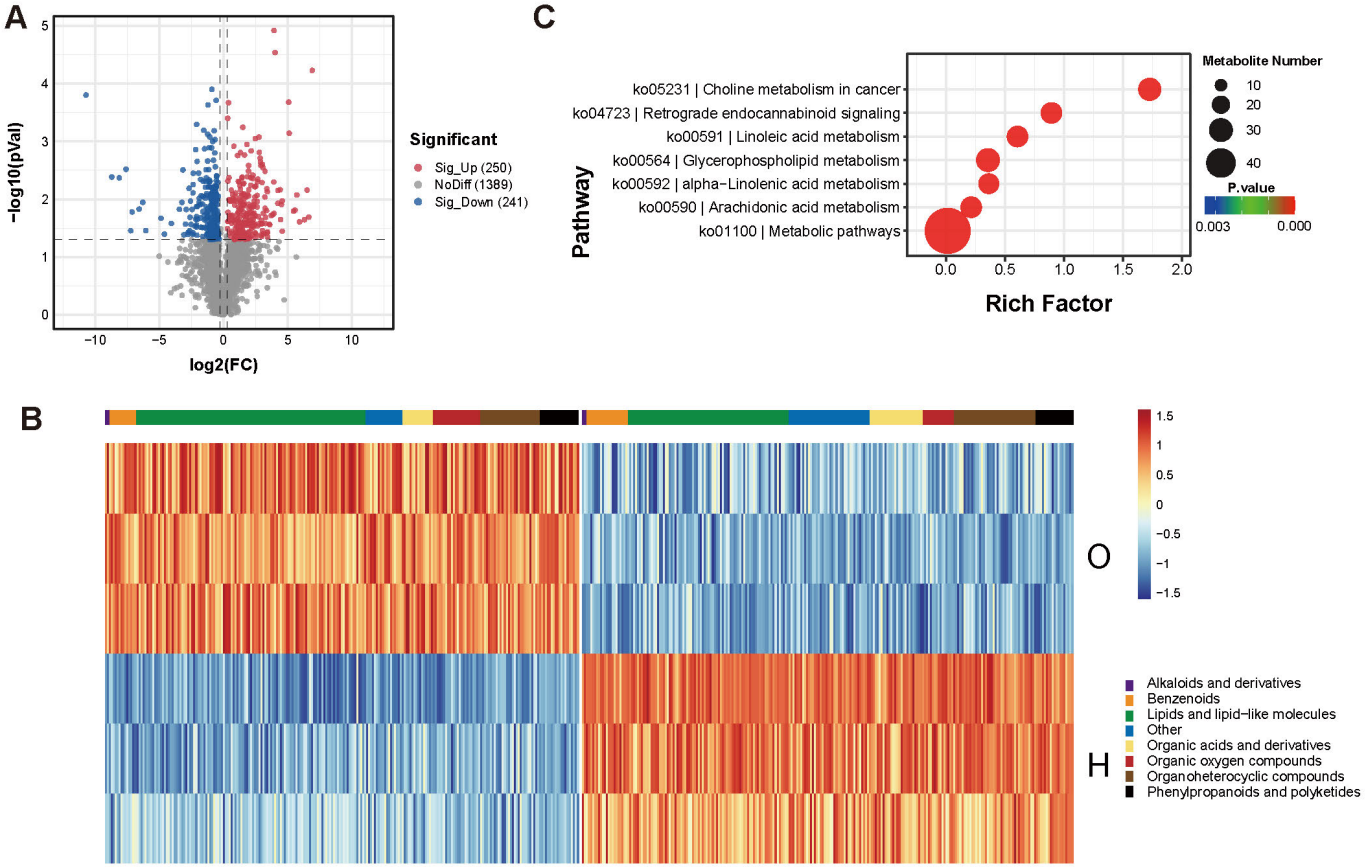
Differentially accumulated metabolites (DAMs) between two cultivars. **(A)** Volcano map of differential metabolites. Red and blue represent metabolites accumulated in the H cultivar and the O cultivar, respectively. **(B)** Heatmap of differentially accumulated metabolites (DAMs) between two cultivars. DAMs were clustered based on their types. **(C)** KEGG analysis of DAMs identified between two cultivars.

Then we compared the differentially accumulated metabolites (DAMs) between the two cultivars. In total, 250 metabolites were highly accumulated in the H cultivar, while 241 metabolites were highly accumulated in the O cultivar (**Figure 2A**). In the Top30 metabolites, the content of flavonoids showed significant differences. For example, flavonoid compounds such as Kaempferol and 2’-Hydroxygenistein 8-C-glucoside were found in much higher quantities in the H cultivar than in the O cultivar. Additionally, Diketene, an important synthetic precursor that can participate in the synthesis of flavonoids, alkaloids, and other secondary metabolites (Marcus and Chan, 1967), was also significantly enriched in the H cultivar (**Figure 2B**).

In contrast, the O cultivar was also rich in some flavonoid metabolites, such as Tectoridin and Isopongaflavone. Moreover, membrane lipid metabolites such as PC (O-16:1/18:0) and PC (O-18:1(11Z)/16:0) were enriched in the O cultivar. Among them, a large number of phosphatidylcholine (PC) compounds, such as PC (18:1(11Z)/18:2 (9Z,12Z)), PC (18:0/22:5), and PC (18:0/20:1), were enriched in the O cultivar. These compounds are important components of cell membranes. Not surprisingly, the metabolomics analysis identified higher levels of Cannabichromene and delta8-Tetrahydrocannabinol in the H cultivar. KEGG analysis showed that DAMs are mainly enriched in Linoleic acid metabolism (ko00591), Glycerophospholipid metabolism (ko00564), and alpha-Linolenic acid metabolism (ko00592) (**Figure 2C**). Among these, a large number of phosphatidylcholine (PC) compounds, such as PC (18:1(11Z)/18:2 (9Z,12Z)), PC (18:0/22:5), and PC (18:0/20:1), were enriched in the O cultivar. These compounds are important components of cell membranes. The results indicate that there are significant differences in the metabolites of the inflorescences between the two cultivars, especially in flavonoids and fatty acids.

### 3.3 Sequencing of the hemp transcriptome using RNA-seq

To further explore the mechanism of the biosynthesis of secondary metabolites, we performed comparative transcriptome analysis of two cultivars. An average of 42 million reads were generated for each biological replicate. After quality control, an average of 41 million reads remained for each replicate Over 75% clean reads were successfully mapped to the reference genome (**Supplementary Table S3**). PCA showed that the two cultivars separated from each other, but replicates were clustered together, suggesting that our transcriptome data is reliable (**Supplementary Figures S1A, B**).

To confirm the reliability of the transcriptome data, seven genes, including flavanone 3-hydroxylase (LOC115702709), dihydroflavonol 4-reductase (LOC115710150), Naringenin-chalcone synthase (LOC115724170), Fatty acid desaturase (LOC115705664), Chlorophyll A-B binding protein (LOC115702322) and Ferredoxin–NADP reductase (LOC115702780), were selected for qPCR. The RT-PCR data showed a similar expression pattern with RNA-seq, which suggests that our RNA-seq data are reliable (**Supplementary Figure S1C**).

**Figure 3:**
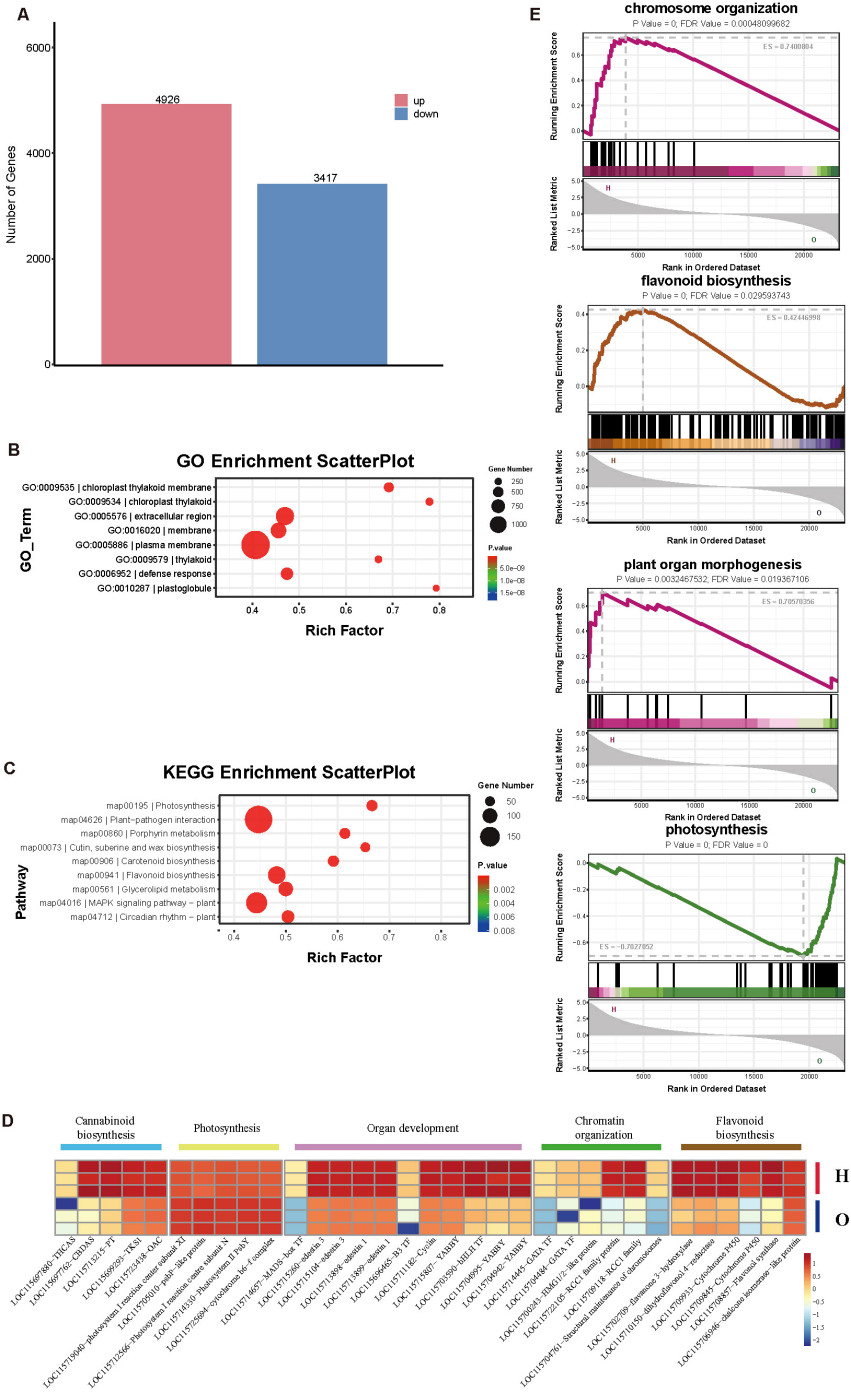
Transcriptional profiling between two cultivars. **(A)** Differentially expressed genes (DEGs) number between the two cultivars. GO **(B)** and KEGG **(C)** enrichment analysis of DEGs. **(D)** Heatmap of selected genes. The Z-score value of the normalized FPKM of the gene was used to draw a heatmap. **(E)** GSEA analysis. The topmost color line represents the Enrichment Score of the gene. The vertical axis indicates the corresponding Running ES. The horizontal axis represents each gene in this gene set, corresponding to the vertical lines similar to barcodes in the second part.

Then we compared the Differentially Expressed Genes (DEGs) between the two cultivars. 4926 genes were upregulated in O cultivar, while 3417 genes were upregulated in H cultivar (**Figure 3A**; **Supplementary Table S4**). Then we annotated the DEGs in the GO and KEGG databases (**Supplementary Figures S1D, E**). The results showed that the number of DEGs in different pathways varied significantly between the two cultivars, indicating that the two cultivars have distinct gene expression regulation patterns at the transcriptional level. In cultivar H, the number of upregulated genes was significantly higher than that in cultivar O in pathways including DNA−templated transcription (GO:0006351), regulation of DNA−templated transcription (GO:0006355), protein binding (GO:0005515) and DNA−binding transcription factor activity (GO:0003700), as well as in pathways related to flavonoid biosynthesis, glycerophospholipid metabolism, fatty acid biosynthesis, and glycolysis/gluconeogenesis. In contrast, cultivar O had a higher number of upregulated genes in items such as protein phosphorylation (GO:0006468) and protein serine/threonine kinase activity (GO:0004674), and in pathways like porphyrin metabolism, photosynthesis, circadian rhythm – plant, and ABC transporters (**Figure 3B**). KEGG enrichment showed that DEGs were mainly enriched in Photosynthesis (map00195), Flavonoid biosynthesis (map00941), MAPK signaling pathway – plant (map04016) pathway (**Figure 3C**). Several key genes in the flavonoid biosynthesis pathway (Liu et al., 2021). For example, chalcone isomerase-like protein (LOC115706946), flavonol synthase (LOC115708857), dihydroflavonol 4-reductase (LOC115710150), and flavanone 3-hydroxylase (LOC115702709) were upregulated in H cultivar (**Figure 3D**). Cytochrome P450 (CYP) is a superfamily of monooxygenases that are widely distributed in plants. These enzymes play a crucial role in the biosynthesis of flavonoids, and compounds significantly influence the diversity of flavonoids. Cytochrome P450 (CYP450), one of the largest enzyme superfamilies in plants, participates in the biosynthesis of diverse secondary metabolites and dictates both their structural diversity and biological activity (Durst and Nelson, 1995; Hansen et al., 2021; Kahn and Durst, 2000). Several family P450 members (LOC115709845 and LOC115709933) were upregulated in the H cultivar.

GSEA (Gene Set Enrichment Analysis) also showed that Plant organ morphogenesis-related items like seed development and specification of floral organ identity were upregulated in H cultivar (**Figure 3E**). Several transcription factors like MADS-box (LOC115714657), B3 (LOC115696465), and YABBY (LOC115704942, LOC115704995, and LOC115715807) genes related to flower and seed development are upregulated in the H cultivar. Storage proteins, like Edestin and Cyclin, were also upregulated in H cultivar (**Figure 3D**). Glandular trichomes are primarily distributed on the sepals of pistillate flowers and the young leaves of the inflorescence (AscensÃO et al., 1999; Livingston et al., 2020; Matías-Hernández et al., 2016). This distribution pattern suggests that flower morphogenesis may be an essential prerequisite for the synthesis of glandular trichomes and secondary metabolites. In the H cultivar, a large number of flower development-related genes exhibit high expression levels, indicating that this cultivar has entered the reproductive growth stage, which in turn initiates the biosynthesis of a substantial amount of secondary metabolites, such as cannabinoids. Additionally, cannabis seeds are rich in lipids and proteins (Aluko, 2017; Garcia et al., 2021). During the seed maturation process of the H cultivar, the significant increase in lipid content is an inevitable outcome. Cannabinoid biosynthesis pathway genes, including OAC (LOC115723438), TKS (LOC115699293), PT (LOC115713215), CBDAS (LOC115697762) also showed high expression in H cultivar (**Figure 3D**).

Genes associated with chromatin organization and chromosome structure were also enriched in the H cultivar (**Figure 3E**). Several chromosome structure factors like Regulator of chromosome condensation (RCC1) family protein (Antonin and Neumann, 2016) (LOC115722105 and LOC115709118) and Structural maintenance of chromosomes (Strunnikov and Jessberger, 1999) (LOC115704761) were highly expressed in H cultivar. The upregulated of these chromosome-related factors suggested that the cell may have undergone significant changes in the biological processes of chromatin structure regulation, gene expression regulation, and the cell cycle (McCord et al., 2020).

While photosynthesis-related genes were upregulated in the O cultivar. For example, cytochrome b6-f complex iron-sulfur subunit 2 (LOC115725694), Photosystem II PsbY (LOC115714330), Photosystem I reaction centre subunit N (LOC115712566, LOC115719040), psbP-like protein 1 (LOC115705010), which were the core proteins of photosynthesis, were upregulated in the O cultivar. These results indicate that photosynthesis remains the predominant bio-process in cultivar O. As a result, the number of glandular trichomes is relatively low, which in turn leads to a reduced content of secondary metabolites such as cannabinoids. Due to the government’s strict anti-drug policies, such as the continuous eradication of high density trichomes cultivars, only low density trichomes cultivars have been preserved and can be cultivated. The transcriptome data indicate a significant difference between the two cultivars, resulting in a notable disparity in their metabolite contents.

### 3.4 Chromatin accessibility profiling

Metabolomic and transcriptomic profiling revealed extensive differences in both metabolites and gene expression between the two cultivars, especially, secondary-metabolite biosynthetic pathways genes and metabolites. We hypothesize that epigenetic regulation contributes to the divergent expression of key pathway genes. Consistently, the transcriptome data also showed significant cultivar-specific differences in the expression of several chromatin-associated genes. To test this, we systematically compare chromatin accessibility in the inflorescences of the two cultivars by using ATAC-seq. Six samples (three biological replicates per cultivar) were sequenced, generating an average of 175–180 million raw reads per sample. After quality filtering, approximately 98–99% of high-quality reads were retained, demonstrating the excellent quality and reliability of the sequencing data. The average Q30 of the clean reads was 93.93% (**Supplementary Table S5**).

**Figure 4:**
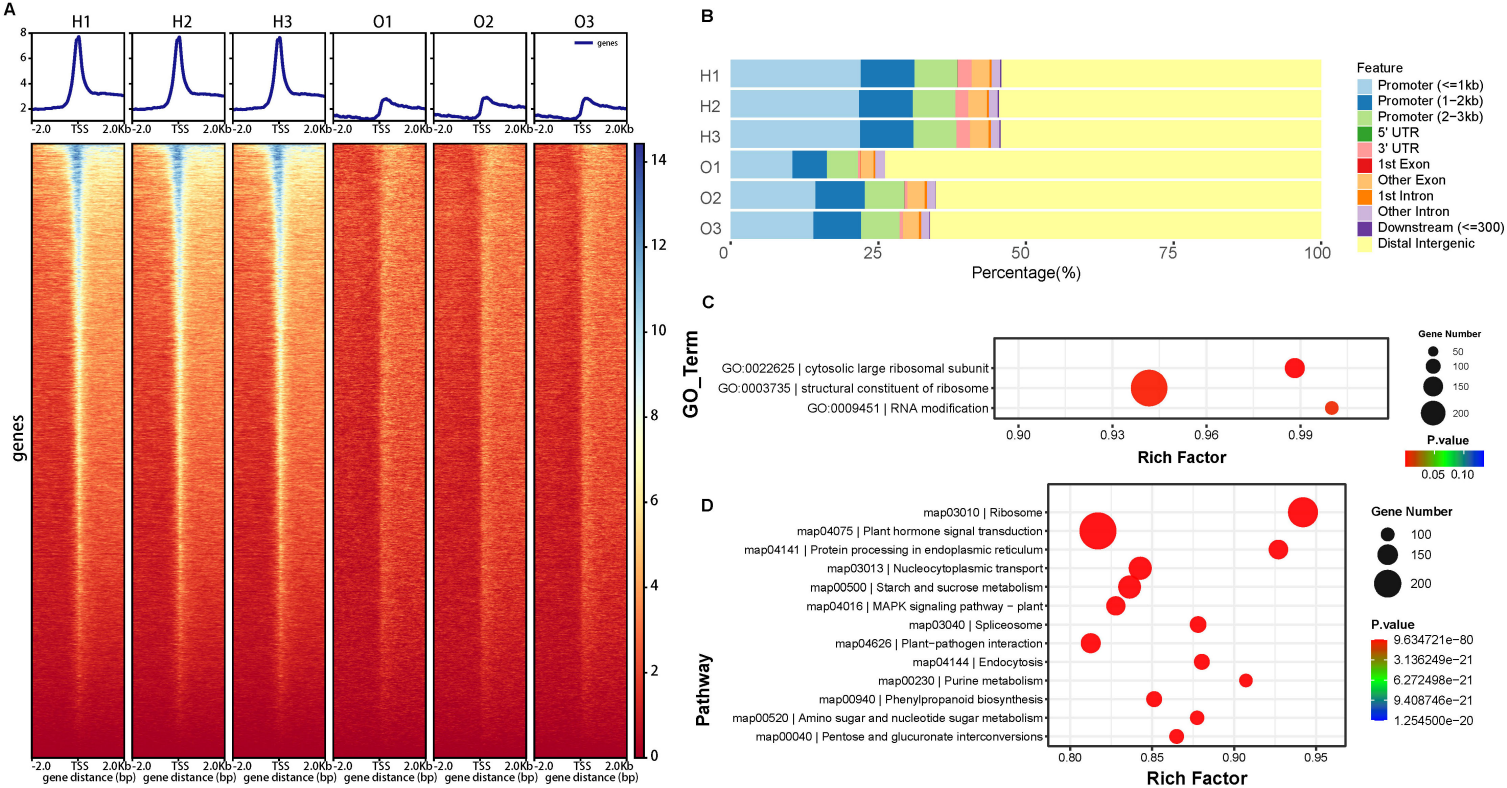
Chromatin accessibility Profiling between two cultivars. **(A)** Enrichment of insertion fragments at the transcription start sites of each sample. The horizontal axis represents the distance from this site to the TSS, and the vertical axis represents the average sequencing depth of this site. **(B)** The proportion of Peak in different genomic functional regions. GO **(C)** and KEGG **(D)** enrichment analysis of different accessibility genes.

The accessible regions identified were predominantly enriched near the Transcription Start Site (TSS) across the genome, indicating the high quality of the ATAC-seq data (**Figure 4A**) (Kawaji et al., 2006). The correlations among the three biological replicates of the H cultivar exceeded 0.95, while the correlations among the three biological replicates of the O cultivar exceeded 0.79. while the correlation between different cultivars is only 0.56 (**Supplementary Figure S2**). These results indicate that there are differences in chromatin accessibility between the two cultivars, and the data showed repeatability and reliability.

Subsequently, we annotated the peaks to genes. The results showed that the vast majority of the peaks were annotated in the distal intergenic regions (about 50% for the H cultivar and about 73% for the O cultivar). The promoters play a crucial role in regulating gene expression. A large number of peaks were annotated in the promoter regions (about 37% for the H cultivar and about 20% for the O cultivar), and the proximal (<1kb) region of the promoter was enriched with more peaks than the distal region (**Figure 4B**). These peaks in the promoter region may affect the promoter driving force. A small number of peaks were annotated in the UTR and gene body regions (introns, exons, and downstream).

DARs and the associated genes (different accessibility genes, DAGs) were detailed in **Supplementary Table S6**. Totally, 400 genes showed high accessibility in the O cultivar, while 10976 genes showed high accessibility in the H cultivar. GO enrichment analysis showed that cytosolic large ribosomal subunit (GO:0022625), structural constituent of ribosome (GO:0003735), and RNA modification (GO:0009451) related genes showed different accessibility between the two cultivars (**Figure 4C**). KEGG annotation showed that many metabolic pathway genes (Starch and sucrose metabolism (map00500) and Phenylpropanoid biosynthesis (map00940)) exhibit chromatin accessibility differences between the two cultivars (**Figure 4D**), which may lead to variations in gene expression within the pathways and subsequently result in differences in metabolite levels. For example, Naringenin-chalcone synthase (LOC115724170 and LOC115712997) and Cytochrome P450 (LOC115709845, LOC115709933), which belong to the flavonoid biosynthesis pathway (Liu et al., 2021), showed high accessibility in the promoter in H cultivar. The transcriptome analysis showed that the DEGs between the two cultivars were enriched in the flavonoid biosynthesis pathway. Our result suggested that chromatin accessibility in these genes might lead to a high expression of these pathway genes in H cultivar. Glycoside hydrolases (Minic, 2008), which are highly involved in the metabolism of various carbohydrates containing compounds present in the plant tissues, showed high accessibility in promoter. These DARs might lead to different starch and sugar contents between the two cultivars. The phenylpropanoid biosynthesis pathway is one of the upstream pathways for cannabinoid synthesis (Docimo et al., 2013). The metabolites of phenylpropanoid compounds (such as shikimic acid, cinnamic acid, etc.) are the key precursor substances for the synthesis of cannabinoids. Phenylalanine is converted into cinnamic acid by Phenylalanine ammonia lyase (PAL). Cannabis homologous gene of *PAL* (LOC115709862) showed high accessibility in the promoter in H cultivar. This might be one of the reasons for the increase in cannabinoid content in the H cultivar.

DARs were also enriched in Plant hormone signal transduction (map04075), MAPK signaling pathway – plant (map04016), indicating that there are differences in the signal pathway transduction of the two cultivars. Promoter of two-component signaling (Lohrmann and Harter, 2002) elements systems genes (Ruszkowski et al., 2013): histidine-containing phosphotransferase protein (LOC115705353) and the regulator (LOC115709125) showed high accessibility in the H cultivar. ABSCISIC ACID-INSENSITIVE 5 (ABI5, LOC115708807) also showed high accessibility in the promoter in the H cultivar than O cultivar. While promoters of many transcription factors (Basic helix-loop-helix, GRAS, and AP2) showed high accessibility in the O cultivar. These results suggested that there are epigenetic differences between the two varieties in the process of hormone signal transduction.

### 3.5 Integration of metabolome, transcriptome, and ATAC-seq data

**Figure 5:**
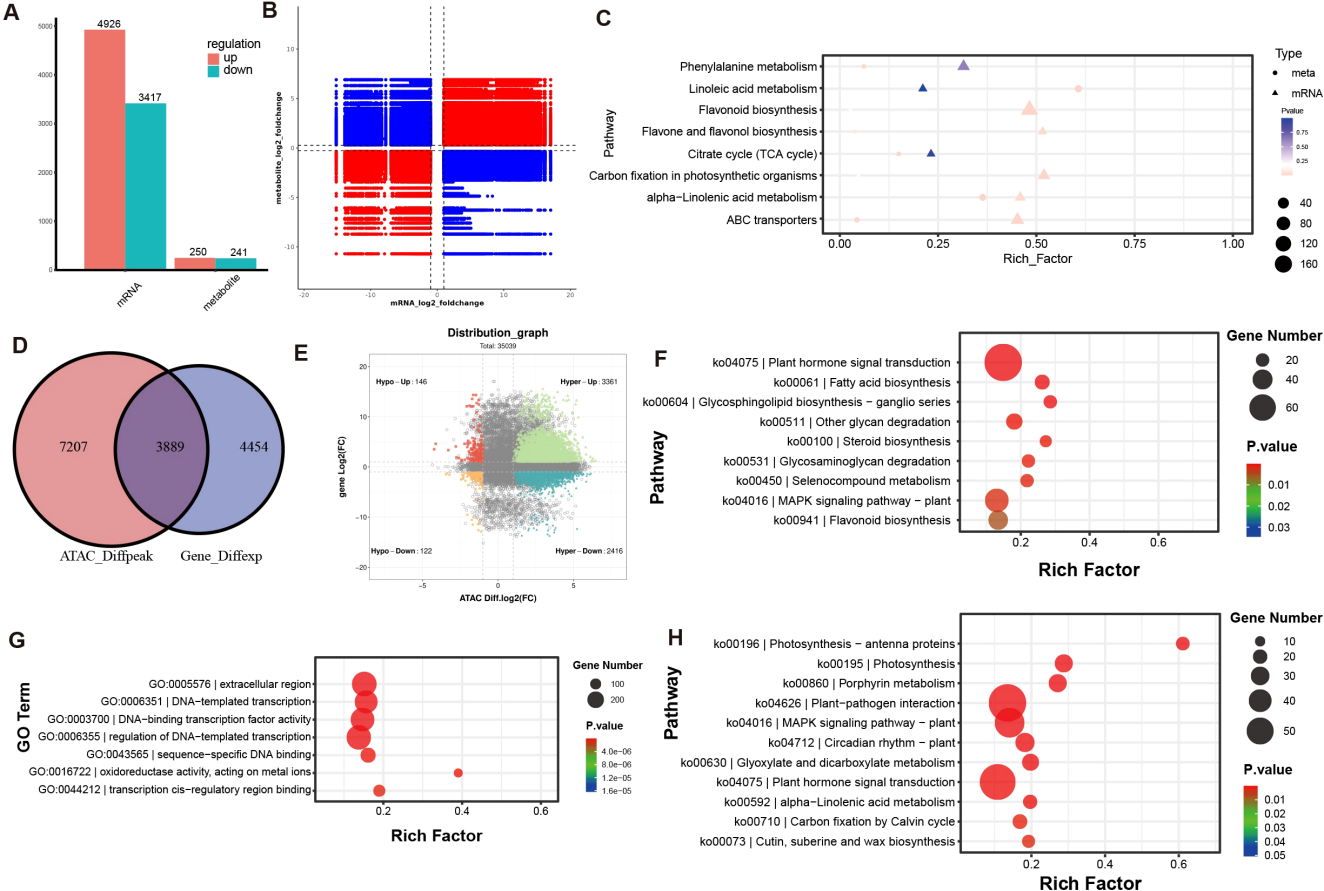
Integration of metabolome, transcriptome, and ATAC-seq. **(A)** Number of DEGs and DAMs. **(B)** Nine-quadrant diagram of metabolome, transcriptome. The horizontal axis represents the mRNA log2 fold change. The vertical axis represents the log2 fold change of the metabolites. **(C)** KEGG enrichment of DEGs and DAMs. **(D)** Venn of different peaks and different genes that identified between two varieties. **(E)** nine-quadrant diagram of transcriptome and ATAC-seq. GO and KEGG enrichment of Hyper-up gene **(F, G)** and Hyper-down **(H)**.

In order to conduct a more comprehensive and integrated analysis of the differences between the two cultivars, we performed integrated transcriptome-metabolism and transcriptome-ATAC-seq analyses. Totally, 250 metabolites and 4,926 genes were highly enriched in the H cultivar, while in the O cultivar, 241 metabolites and 3,417 genes were highly accumulated (**Figure 5A**). The nine-quadrant diagram divided the differentially expressed genes and metabolites into gene sets that promote metabolite synthesis and gene sets that inhibit metabolite synthesis (**Figure 5B**). Pathways such as alpha-Linolenic acid metabolism, ABC transporter, Carbon fixation in photosynthetic organisms, and Flavonoid biosynthesis all have differentially expressed genes and metabolites (**Figure 5C**).

**Figure 6:**
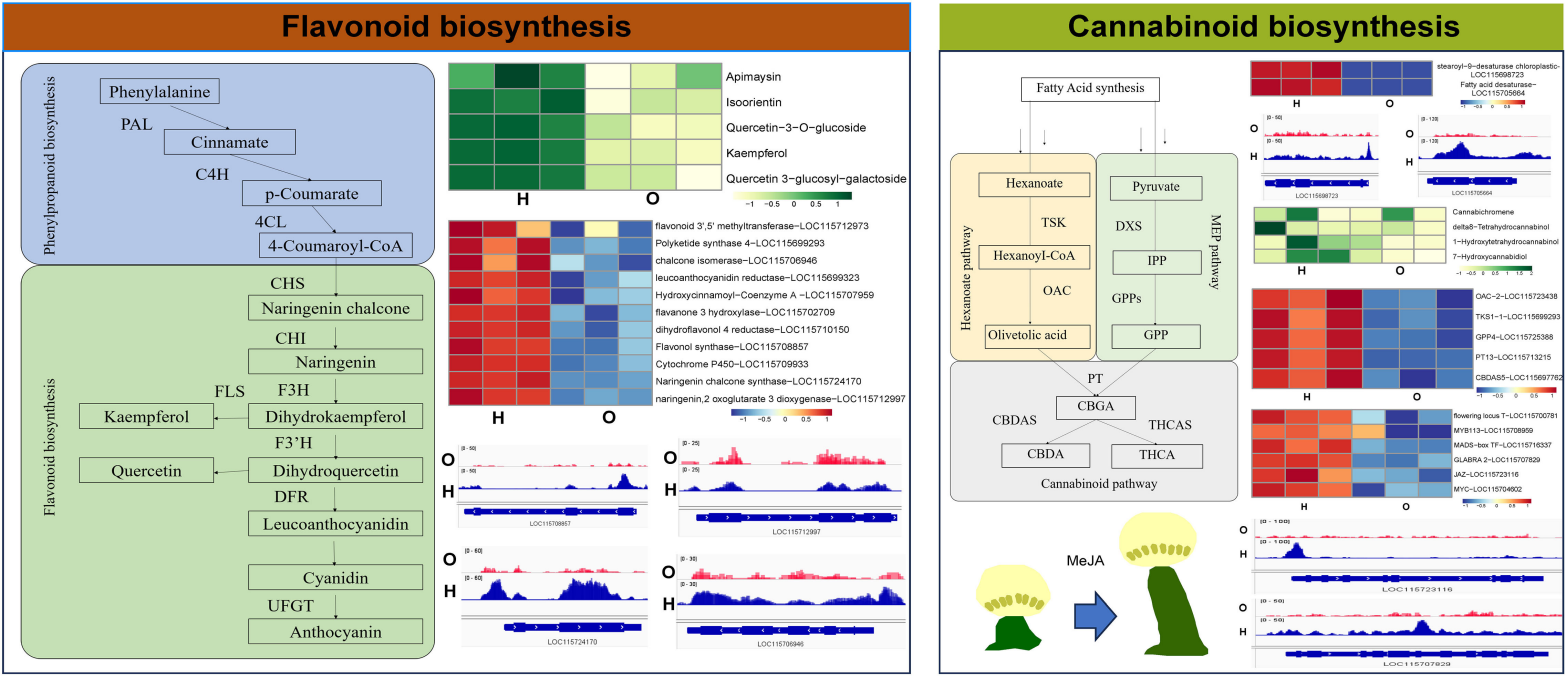
Comprehensive analysis of the flavonoid synthesis pathway and the cannabinoid synthesis pathway.

A total of 8,343 DEGs and 11,096 DAGs were subjected to integrative analysis, revealing 6,045 genes whose chromatin accessibility levels were significantly correlated with their transcript abundances (**Figure 5D**, **Supplementary Table S7**). Given the markedly fewer DAGs detected in the O cultivar by ATAC-seq, we focused on genes in the H cultivar that exhibited increased chromatin accessibility yet displayed divergent expression patterns. Specifically, 3,361 genes (Hyper-up) showed enhanced chromatin accessibility within their gene bodies and were transcriptionally up-regulated in H cultivar (**Figure 5E**). Conversely, 2,416 genes (Hyper-down) displayed elevated chromatin accessibility but were transcriptionally down-regulated in the H cultivar. Subsequent GO and KEGG enrichment analyses revealed that Hyper-up genes were significantly enriched in pathways such as plant hormone signal transduction (ko04075), fatty acid biosynthesis (ko00061), and flavonoid biosynthesis (ko00941) (**Figure 5F**). These genes were also over-represented in GO terms including DNA-templated transcription (GO:0006351), DNA-binding transcription factor activity (GO:0003700), and transcription cis-regulatory region binding (GO:0044212) (**Figure 5G**). In contrast, Hyper-down genes were preferentially enriched in photosynthesis (ko00195), circadian rhythm—plant (ko04712), and alpha-linolenic acid metabolism (ko00592) (**Figure 5H**), and response to chitin (GO:0010200), response to wounding (GO:0009611), and DNA−binding transcription factor activity (GO:0003700) (**Supplementary Figure S3A**). Our results showed that in addition to genes related to cannabinoid biosynthesis and genes related to the initiation/development of glandular hairs, other genes such as floral transition and genes regulating chromatin opening may also affect the content of cannabinoid (**Figure 6**).

We subsequently performed a systematic evaluation of flavonoid and cannabinoid biosynthetic pathways in two hemp cultivars. Metabolomics revealed that flavonoids such as kaempferol, isoorientin, and quercetin-3-O-glucoside accumulated to significantly higher levels in cultivar H than in cultivar O. Transcriptomic profiling further showed that key flavonoid biosynthetic genes, including flavonoid 3′,5′-methyltransferase (LOC115712973), chalcone isomerase (CHI, LOC115706946), chalcone synthase (LOC115724170) and flavonol synthase (LOC115708857) were markedly up-regulated in cultivar H. ATAC-seq data showed that chromatin accessibility within 2 kb upstream of the transcription start sites of these genes was significantly increased in cultivar H. Increased chromatin accessibility at promoter regions enables transcription factors, enhancers, and other regulatory elements to bind to promoter motifs, thereby activating or repressing the expression of downstream genes (Fraser et al., 2021; Schoenfelder and Fraser, 2019; Starks et al., 2019). Collectively, these findings suggest that an open chromatin state at the promoters of key flavonoid biosynthetic genes in cultivar H enhances their transcriptional activity, thereby driving efficient flavonoid accumulation.

A systematic comparison of the cannabinoid biosynthetic pathways in the two cultivars revealed that cannabinoids such as cannabichromene, Δ8-tetrahydrocannabinol and 1-hydroxytetrahydrocannabinol accumulate to higher levels in cultivar H than in cultivar O. Transcriptomic profiling showed that the key biosynthetic enzyme genes—OAC (LOC115723438), TKS (LOC115699293) and CBDAS (LOC115697762)—are significantly up-regulated in cultivar H. ATAC-seq showed that peaks disturbed in the gene bodies or promoters of these genes, however, peak intensities did not differ significantly between the two cultivars (**Supplementary Figure S3C**). This suggests that chromatin accessibility contributes to the expression of the genes in cannabinoids biosynthetic pathway but is unlikely to be the key driver of the observed variation in cannabinoid content between the cultivars. Greater chromatin accessibility at the promoter exposes transcription-factor binding sites, facilitating their rapid occupancy and driving gene transcription. Liu et al. (2021) identified three novel transcription factors that can bind to the promoter of the THCAS gene and alter its expression. The peaks in the promoter might be the prerequisite for this transcriptional regulation. Our data showed that the peak located on the promoter of OAC (LOC115723438), TKS (LOC115699293) and CBDAS (LOC115697762) might be the key regions for cannabinoid biosynthesis regulation for further study.

Cannabinoid biosynthesis is strongly dependent on fatty-acid-derived precursors (Luo et al., 2019; Stout et al., 2012). Quantitative results demonstrated that fatty-acid metabolic pathway genes like fatty-acid desaturase (LOC115705664), stearoyl-9-desaturase (LOC115698723), and acetyl-CoA carrier (LOC115720238) showed increased chromatin accessibility at their promoters in cultivar H, leading to significantly higher expression. It indicated that chromatin accessibility alters the content of cannabinoids by influencing the biosynthesis genes of cannabinoid precursors. At the same time, these fatty acids are also important components of cannabis seeds, indicating that the development of cannabis seeds may also be regulated by epigenetics (Moreno-Pérez et al., 2021; Zhang et al., 2024, Zhang et al., 2025). Cannabinoids are synthesized and stored in glandular trichomes; therefore, trichome density directly determines cannabinoid content. Trichome development has been extensively studied in tomato, Tobacco, and *Arabidopsis*. Genes such as SlMXI (Lu and Zhu, 2022), NtMYC2a/b (Sui et al., 2021), GLABRA1 (GL1) (Maes et al., 2008), and GLABRA2 (GL2) (Ohashi et al., 2002; Wang et al., 2010) are recognized as key regulators of glandular trichome development. The core trichome regulator GLABRA 2 (Ohashi et al., 2002; Rerie et al., 1994) (LOC115707829) is markedly up-regulated in cultivar H and exhibits elevated promoter accessibility. It may be the key reason for the difference in the number of glandular trichomes between the two cultivars. Previous reports have shown that exogenous Methyl Jasmonate (MeJA) increases the density of trichomes. Our data showed that the key MeJA-responsive genes like JAZ (LOC115723116) and MYC (LOC115704602) are also highly expressed in cultivar H with significantly enhanced chromatin openness at their promoters (Liu et al., 2023). Trichomes are predominantly enriched on the bracts of female flowers and leaves of the inflorescence, linking trichome development tightly to flowering (Hesami et al., 2023; Livingston et al., 2020; Sutton et al., 2023). In cultivar H, the flowering integrator FLOWERING LOCUS T (Dowling et al., 2024) (LOC115700781) and the floral-identity transcription factors MYB113 (LOC115708959), MADS-box (LOC115716337), and YABBY (LOC115704995) are all significantly up-regulated, accompanied by increased chromatin accessibility at their promoters.

This study explored the regulatory mechanism of secondary metabolite synthesis in industrial hemp from the perspective of ‘chromatin opening - transcription - metabolism’. However, it is worth noting that the differences at the genomic level between the two varieties might be an important reason for the differences between them. For example, A large number of SNPs are present in the genomes of Marijuana and hemp, which leads to differences in gene expression between the two species and these differences are not limited to genes related to the cannabinoids biosynthesis gene like THCAS (Sawler et al., 2015). Laverty et al. (2019) found that THCAS and CBDAS contained large retrotransposon-rich regions that are highly nonhomologous between drug- and hemp-type alleles. Therefore, in the future, comparing the differences in the genomes of the O and H varieties may reveal more factors that affect the synthesis of cannabinoids.

Collectively, the chromatin accessibility of genes in the cannabinoid biosynthesis pathway is not the cause of the difference in cannabinoid content between the two cultivars. Instead, the elevated cannabinoid accumulation in cultivar H appears to be driven primarily by an enhanced supply of precursors (up-regulated fatty-acid metabolism) and increased trichome abundance. In contrast, the augmented expression of genes involved in precursor synthesis, trichome development, jasmonate signaling, and flowering control is strongly associated with epigenetic activation, manifested as heightened chromatin accessibility at their respective promoters.

## Data Availability

The datasets presented in this study can be found in online repositories. The names of the repository/repositories and accession number(s) can be found below: https://www.ncbi.nlm.nih.gov/, PRJNA1311642.
