# Supplementary material for: Chromatin accessibility directly governs flavonoid biosynthesis and indirectly orchestrates cannabinoid production in Cannabis
Source: Front Plant Sci. 2026 Jan 19;16:1687700. doi: 10.3389/fpls.2025.1687700 (PMC12861918; doi:10.3389/fpls.2025.1687700)
Supplement: Supplementary file 1 [file Image1.pdf]

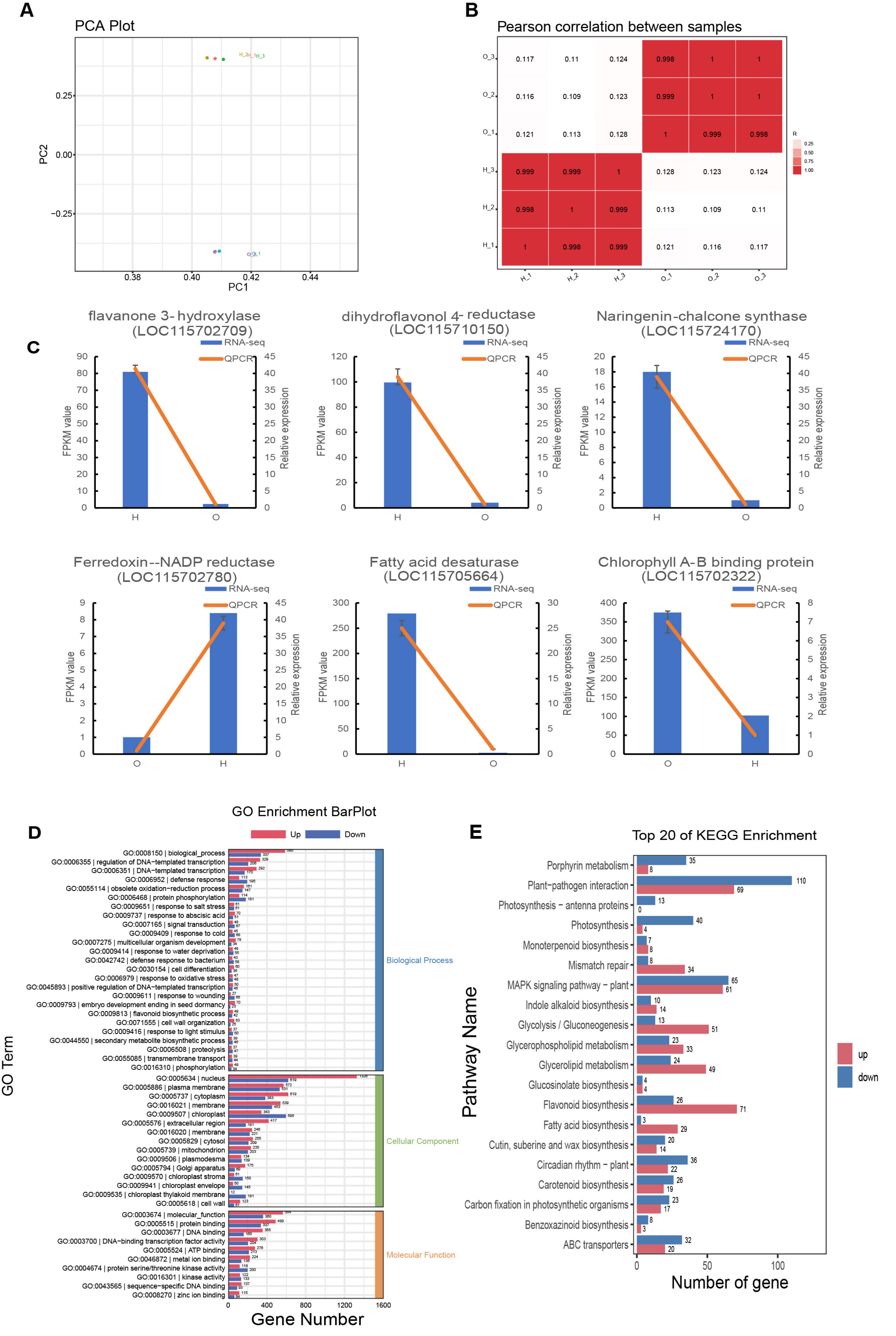

Supplementary figure1: summary of transcriptome data. A. PCA of all the samples. **B.** Pearson correlation between samples. **C.** RT-PCR of the mRNAs to validate data reliability. RT-PCR analysis and Transcriptome data of 6 genes. The RT-PCR data are shown in the orange column, while the Transcriptome data are represented by the blue line. GO **(D)** and KEGG **(E)** of different expressed genes.
