# Supplementary figures and images for: Chromatin accessibility directly governs flavonoid biosynthesis and indirectly orchestrates cannabinoid production in Cannabis
Source: Front Plant Sci. 2026 Jan 19;16:1687700. doi: 10.3389/fpls.2025.1687700 (PMC12861918; doi:10.3389/fpls.2025.1687700)

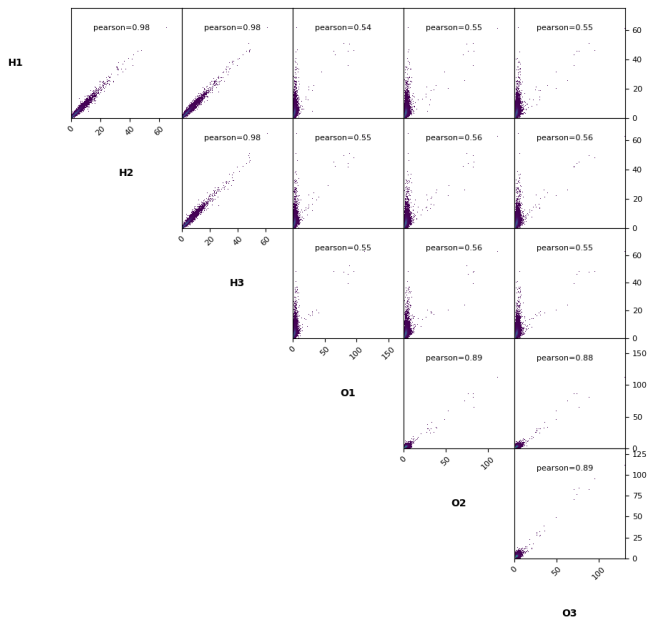

Supplementary figure2: Pearson correlation of average scores per transcript.

Supplement: Supplementary file 2 [file Image2.pdf]
