# Supplementary material for: Chromatin accessibility directly governs flavonoid biosynthesis and indirectly orchestrates cannabinoid production in Cannabis
Source: Front Plant Sci. 2026 Jan 19;16:1687700. doi: 10.3389/fpls.2025.1687700 (PMC12861918; doi:10.3389/fpls.2025.1687700)
Supplement: Supplementary file 3 [file Image3.pdf]

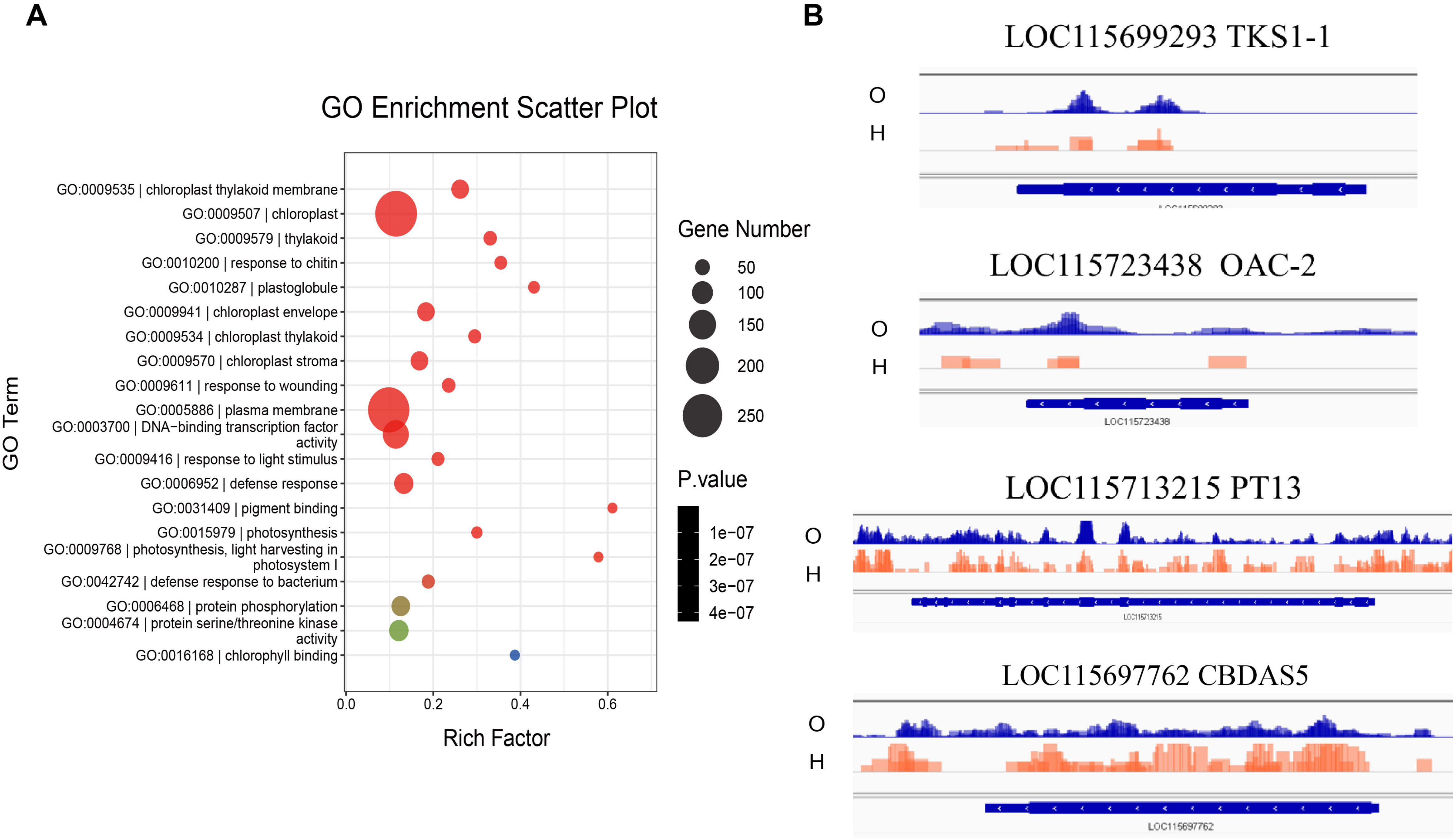

Supplementary figure3: Integration of metabolome, transcriptome, and ATAC-seq data.

A. GO enrichment of Hyper-down gene.

B. Peak identified in cannabinoid biosynthesis pathway genes.
